# The correlation between enlarged perivascular spaces and cognitive impairment in Parkinson’s disease and vascular parkinsonism

**DOI:** 10.1186/s12883-022-02819-7

**Published:** 2022-07-29

**Authors:** Yu Tu, Wenyan Zhuo, Jiewei Peng, Rong Huang, Baizhu Li, Yuqi Liu, Chengtao Zhang, Xiuli Zeng, Li’an Huang

**Affiliations:** 1 Department of Neurology, Zhuhai People’s Hospital (Zhuhai Hospital Affiliated With Jinan University), No.79 KangNing Road, Zhuhai, 519000 Guangdong Province China; 2 Department of Neurology, The First Affiliated Hospital, Jinan University, 601 Huangpu Dadao West, Guangzhou, 510632 Guangdong Province China

**Keywords:** Enlarged perivascular spaces, Vascular parkinsonism, Parkinson’s disease, Cognitive impairment, White matter hyperintensity

## Abstract

**Introduction:**

The widespread use of brain magnetic resonance imaging (MRI) has revealed the correlation between enlarged perivascular spaces (EPVS) and cognitive impairment (CI). However, few studies have examined the correlation between MRI-visible EPVS and CI in patients with Parkinson’s disease (PD) and vascular parkinsonism (VaP). This study explored how the number and main location of EPVS in PD and VaP are correlated with the occurrence of CI in these diseases to provide radiology markers and other evidence for early clinical diagnosis in a Chinese cohort.

**Methods:**

Clinical data were prospectively collected from 77 patients: 26 patients clinically diagnosed with PD or probable PD, 19 patients clinically diagnosed with VaP, and 32 control subjects with normal cognitive function and no stroke or parkinsonism. The patients with PD and VaP were divided into a CI group and a no CI (NCI) group according to the Montreal Cognitive Assessment Beijing version (MoCA-BJ). The relevant clinical data were statistically analysed.

**Results:**

The centrum semiovale (CSO)-EPVS, lacunes, Fazekas scores, global cortical atrophy scale (GCA) scores, Koedam posterior atrophy visual scale (KS) scores, and medial temporal atrophy (MTA) scores were higher in the PD-CI and VaP-CI groups than in the control group (*adjusted P* < 0.017). The number of basal ganglia (BG)-EPVS in the VaP group was higher than that in the PD and control groups (*adjusted P* < 0.017). BG-EPVS, Fazekas scores, GCA scores, KS scores, and MTA scores were higher in the VaP-CI group than in the PD-CI group (*adjusted P* < 0.017). Multivariate logistic regression analysis showed that the differences in BG-EPVS and Fazekas scores were not significant between PD-CI and VaP-CI patients (*P* > 0.05).

**Conclusion:**

VaP-CI results from multiple factors and is significantly associated with BG-EPVS, lacunes, white matter hyperintensities and brain atrophy. BG-EPVS can be used as an imaging marker to distinguish VaP-CI from PD-CI.

## Introduction

Parkinsonism is a common neurological disease that includes typical Parkinson’s disease (PD), atypical parkinsonism, and secondary parkinsonism. Vascular parkinsonism (VaP) is a type of secondary parkinsonism that is often combined with high-risk factors for various cerebrovascular diseases, and the main lesion sites include the subcortical white matter (WM), basal ganglia (BG), thalamus, and midbrain [1]. Benítez-Rivero et al. [2] found that compared with PD patients or healthy controls, VaP patients showed more severe cognitive decline and impaired attention. Typical PD is a common chronic degenerative neurodegenerative disease, and cognitive decline is one of the most common and disabling nonmotor features of PD, with a higher incidence of cognitive impairment (CI) in men aged 60 to 80 years [3].

Recently, as an important basis of vascular cognitive impairment, cerebellar small vessel disease (CSVD) has been shown to be prominent in cognitive impairment in VaP, although its role in CI in PD has been less studied. As an imaging manifestation of CSVD, the relationship between enlarged perivascular spaces (EPVS) and cognitive dysfunction of VaP and PD has attracted considerable attention. Lee et al. [4] found that BG-EPVS played no significant role in dopaminergic innervation revealed by dopamine transporter positron emission tomography (DAT-PET) and made little contribution to clinical parkinsonism but was closely related to the CI (MoCA-Korea score assessment) caused by parkinsonism. The increase in the number of BG-EPVS is positively correlated with the severity of CI in PD patients [5]. Changes in BG-EPVS detected on magnetic resonance imaging (MRI) scans are not correlated with extrapyramidal symptoms or CI [6]. The results of the above studies are inconsistent, and relevant reports of EPVS in VaP and VaP-CI are rare. This study investigated the correlations between the location (BG, centrum semiovale [CSO]) and the number of EPVS with VaP and PD and the occurrence of CI in these diseases and their clinical significance.

## Patients and methods

### Patients

PS patients hospitalized at the Department of Neurology and the Department of Cerebrovascular Disease, Zhuhai People’s Hospital, China, from August 2020 to July 2021 were the study subjects. Parkinsonism and PD were diagnosed in accordance with the Movement Disorder Society (MDS) Clinical Diagnostic Criteria for Parkinson’s disease [7]. Patients with the VaP subtype met the diagnostic criteria for VaP proposed by Zijlmans [8] in 2004. There may be two types of onset of VaP: an acute onset, possibly associated with basal ganglionic infarctions, and an insidious onset, associated more with diffuse subcortical white matter lesions. In our study, all VaP patients had an insidious onset. The inclusion criteria for this study were as follows: (A) consciousness and stable vital signs; (B) age > 18 years and ≤ 85 years; and (C) reports from 1–2 family members who had accompanied the patients for > 3 years confirming that the patient had normal cognitive function and mental behaviour before disease onset. The exclusion criteria were as follows: (A) confirmed transient ischaemic attack (TIA) and acute stroke; (B) cognitive impairment caused by other diseases; (C) an inability to cooperate with cognitive examination and MRI examination; and (D) previous severe depression, suicidality, drug and alcohol abuse, or mental disorders. The endpoint outcome was death, including cardiovascular death, cerebrovascular death, sudden death, or other cause of death.

Fifty patients with parkinsonism were included. One patient with multiple-system atrophy–P type, 1 with drug-induced parkinsonism, and 3 patients with parkinsonism–unclassified type were excluded. Finally, 45 patients were included in the final analysis. Among them, 26 patients were clinically diagnosed with PD or probable PD, and 19 patients were diagnosed with VaP. Thirty-two patients who had normal cognitive function and no stroke or parkinsonism constituted the control group. Among them, 15 hospitalized patients had acute facial neuritis, 5 patients had migraine, 3 patients had benign paroxysmal positional vertigo, 4 patients had somatization disorder, 3 patients had peripheral nerve disease, 1 patient had restless legs syndrome, and 1 patient had vagal reflex syncope.

This clinical study was reviewed and approved by the Ethics Committee of Zhuhai People’s Hospital. All enrolled patients signed a written informed consent form. This study was registered with the China Clinical Trial Registry under registration number ChiCTR2000038819.

### Clinical assessment

The baseline clinical data of the enrolled patients were collected, and age, sex, education levels, duration of onset to enrolment, and other indicators were measured, such as substantia nigra-transcranial sonography (SN-TCS) examination. The scale assessment was completed within 3–5 days after admission. Cognitive function was assessed using the MoCA-BJ scale. PD and VaP patients completed the evaluation of the Unified Parkinson’s Disease Rating Scale (UPDRS) and Hoehn-Yahr staging (H-Y staging).

### MRI evaluation

All patients underwent an examination using a 3.0 T MRI by GE, USA, within 2–5 days after admission. The slice thickness was 5 mm, and the spacing was 0.4 mm. According to the criteria for EPVS [9], the maximum diameter was measured on T2-weighted MRI (T2WI), and the lesion diameter was generally > 2 mm [10]. The numbers of EPVS in the BG, CSO, brainstem, and bilateral hippocampus were recorded, and the MRI T2WI slice with the most EPVS was counted and scored. For BG-EPVS and CSO-EPVS, the number on the side with the most severe lesion was used. EPVS can also be divided into 3 types according to the lesion site. Type I is the BG type, Type II is the cerebral white matter type or cerebral hemisphere type, which is the most common type, and Type III is the mesencephalic type [11].

MRI was used to assess the presence and severity of different signs of CSVD, including lacunes, cerebral microbleeds (CMBs), and white matter hyperintensities (WMHs). White matter lesions were assessed according to the Fazekas [12] scale (0–6 points) [13] on the basis of visual assessment of both periventricular (0 = absent, 1 = caps or pencil lining, 2 = smooth halo, and 3 = irregular periventricular hyperintensities extending into deep white matter) and subcortical areas (0 = absent, 1 = punctuate foci, 2 = beginning confluence of foci, and 3 = large confluent areas). The total Fazekas score was calculated by adding the periventricular and subcortical scores. The Fazekas scale score represents the severity of WMH. 3D Slicer (version 4.11.0) software was used to reconstruct the axial position and obtain coronal MRI images. Brain atrophy was assessed by axial and coronal MRI images. Cortical atrophy was assessed by global cortical atrophy scale (GCA) scores [14]. The posterior cingulate gyrus and precuneus were scored by Koedam posterior atrophy visual scale (KS) scores [15]. Medial temporal atrophy (MTA) scores [16] were determined for atrophy of the medial temporal lobe. GCA scores, KS scores, and MTA scores were combined to assess the severity of brain atrophy.

### Statistics

Data analysis was conducted with IBM SPSS Statistics 24.0 software. When the variables conformed to a normal distribution, the mean ± standard deviation is used for statistical descriptions; otherwise, the variables are described with the median (quartile) [M (p25-p75)]. Nonparametric data were analysed using Kruskal–Wallis rank-sum tests. The adjusted *P* < 0.05/3 = 0.017 was significantly different between patients in the PD, VaP, and control groups or patients in the PD-CI, VaP-CI, and control groups. Countable data are presented as a ratio or percentage (%), and comparisons between groups were performed using the χ^2^ test. The PD-CI group and VaP-CI group assignment was used as the dependent variable, and patient data that were significantly different between the groups were used as the independent variables for binary multivariate logistic regression analyses. A receiver operating characteristic (ROC) curve analysis was performed using MedCalc 19.1 to evaluate the potential use of BG-EPVS, Fazekas scores, and GCA scores to predict PD-CI and VaP-CI. Indicators with predictive ability were extracted from the ROC curve analysis. *P* < 0.05 was considered statistically significant.

## Results

### Study participants and baseline characteristics in PD and VaP patients

**Fig. 1:**
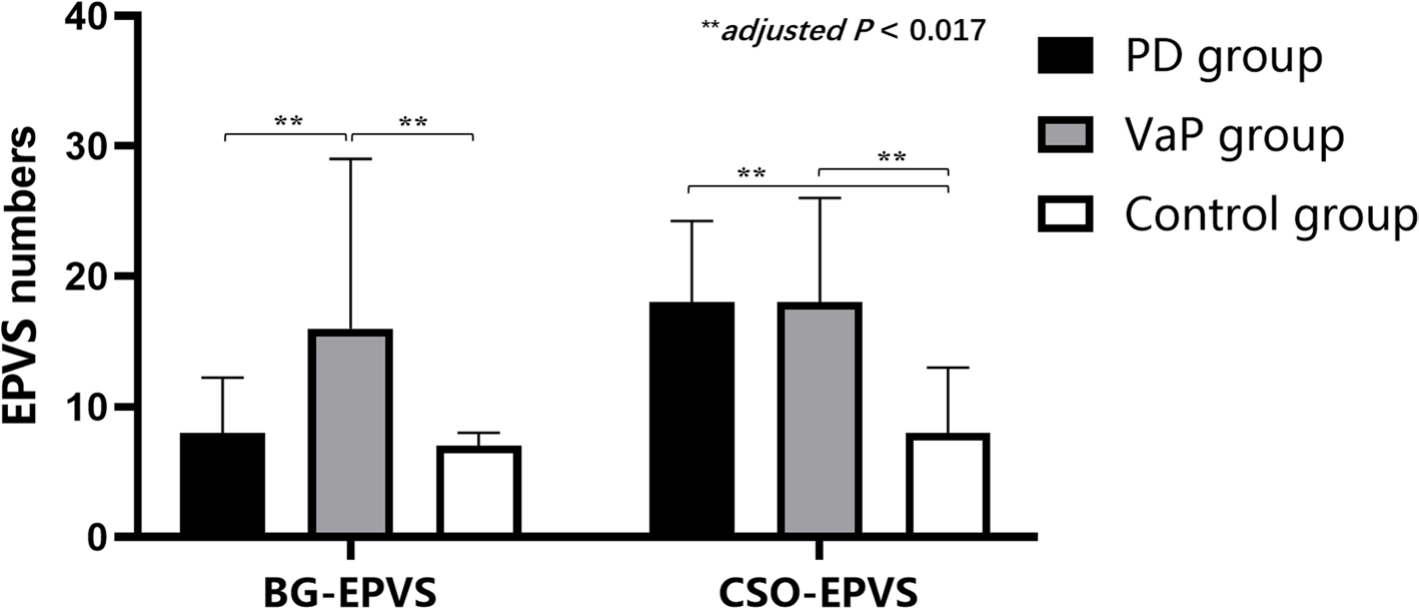
Comparison of the location and number of EPVS among the PD, VaP, and control groups. BG-EPVS in the VaP group were more numerous than in the PD and control groups *(adjusted P* < 0.017). The CSO-EPVS in the PD and VaP groups was higher than that in the control group (*adjusted P* < 0.017)

Age, the rate of a history of diabetes mellitus and stroke, CSO-EPVS, lacunes, Fazekas scores, GCA scores, KS scores, and MTA scores in the PD and VaP groups were higher than those in the control group (*adjusted P* < 0.017). A history of hypertension and BG-EPVS in the VaP group was higher than that in the control group, while educational levels in the VaP group were lower than those in the control group (*adjusted P* < 0.017). In the PD and VaP groups, age, BG-EPVS, the percentage of patients with EPVS I as the main type, Fazekas scores, CMBs, GCA scores, MTA scores, H-Y stage, and UPDRS-III scores of “OFF” in the VaP group were higher than those in the PD group (*adjusted P* < 0.017). SN-TCS positivity and the duration of onset to enrolment between the VaP and PD groups did not reach statistical significance (*adjusted P* > 0.017) (Table 1 and Fig. 1).

**Table 1:** Study participants and baseline characteristics in the PD, VaP, and control groups; *M (P25-P75)*

Variable	PD group (*n* = 26)	VaP group (*n* = 19)	Control group (*n* = 32)	*P* values
Male, n (%)	15 (57.70)	14 (73.70)	19 (59.40)	0.496
Age, years	62.00 (57.50–69.25)^△^	75.00 (69.00–80.00)^△#^	48.50 (37.50–57.00)	< 0.001^***^
Hypertension, n (%)	11 (42.30)	12 (63.20)^△^	8 (25.00)	0.026^*^
Diabetes mellitus, n (%)	6 (23.10)^△^	6 (31.60)^△^	0 (0.00)	0.005^**^
History of stroke, n (%)	5 (19.20)^△^	7.00 (36.80)^△^	0 (0.00)	0.002^**^
History of CAD, n (%)	3 (11.50)	3.00 (15.80)	0 (0.00)	0.086
Cigarette smoking, n (%)	1 (3.80)	4.00 (21.10)	8 (25.00)	0.087
Alcoholism history, n (%)	5 (19.20)	1.00 (5.30)	4 (12.50)	0.385
Education levels: completed secondary school or more, n (%)	20 (76.92)	11.00 (57.89)^△^	31 (96.90)	0.003^**^
BG-EPVS numbers	8.00 (5.50–12.25)	16.00 (11.00–29.00)^△#^	7.00 (4.00–8.00)	< 0.001^***^
CSO-EPVS numbers	18.00 (10.50–24.25)^△^	18.00 (10.00–26.00)^△^	8.00 (4.25–13.00)	< 0.001^***^
EPVS I as the main type, n (%)	4 (15.40)	10 (52.60) ^#^	10 (31.30)	0.029^*^
Fazekas scores	0.00 (0.00–0.00)^△^	1.00 (1.00–3.00)^△#^	0.00 (0.00–0.00)	< 0.001^***^
CMBs numbers	0.00 (0.00–1.00)	1.00 (0.00–5.00)^#^	-	< 0.001^***^
GCA scores	1.00 (1.00–2.00)^△^	3.00 (2.00–3.00)^△#^	0.00 (0.00–0.00)	< 0.001^***^
KS scores	2.50 (1.75–3.00)^△^	3.00 (2.00–3.00)^△^	0.00 (0.00–0.00)	< 0.001^***^
MTA scores	1.00 (0.00–2.00)^△^	2.00 (1.00–3.00)^△#^	0.00 (0.00–0.00)	< 0.001^***^
Lacunes numbers	0.00 (0.00–3.00)^△^	1.00 (0.00–4.00)^△^	0.00 (0.00–0.00)	< 0.001^***^
UPDRS-III scores “OFF”	35.00 (20.00–45.00)	50.00 (33.00–55.00)^#^	-	0.005^**^
H-Y stages	3.00 (1.75–4.00)	3.00 (3.00–4.00)^#^	-	0.003^**^
SN-TCS positive, n (%)	15 (57.69)	7 (36.84)	-	0.167
Duration of onset to enrolment, years	1.00 (0.50–3.00)	4.00 (1.00–6.00)	-	0.087

^*^P < 0.05, ^**^P < 0.01, ^***^P < 0.001. Compared to the PD group, ^#^*adjusted P* < 0.017. Compared to the control group, ^△^*adjusted P* < 0.017

### Difference analysis of clinical characteristics of CI and NCI in PD and VaP patients

**Fig. 2:**
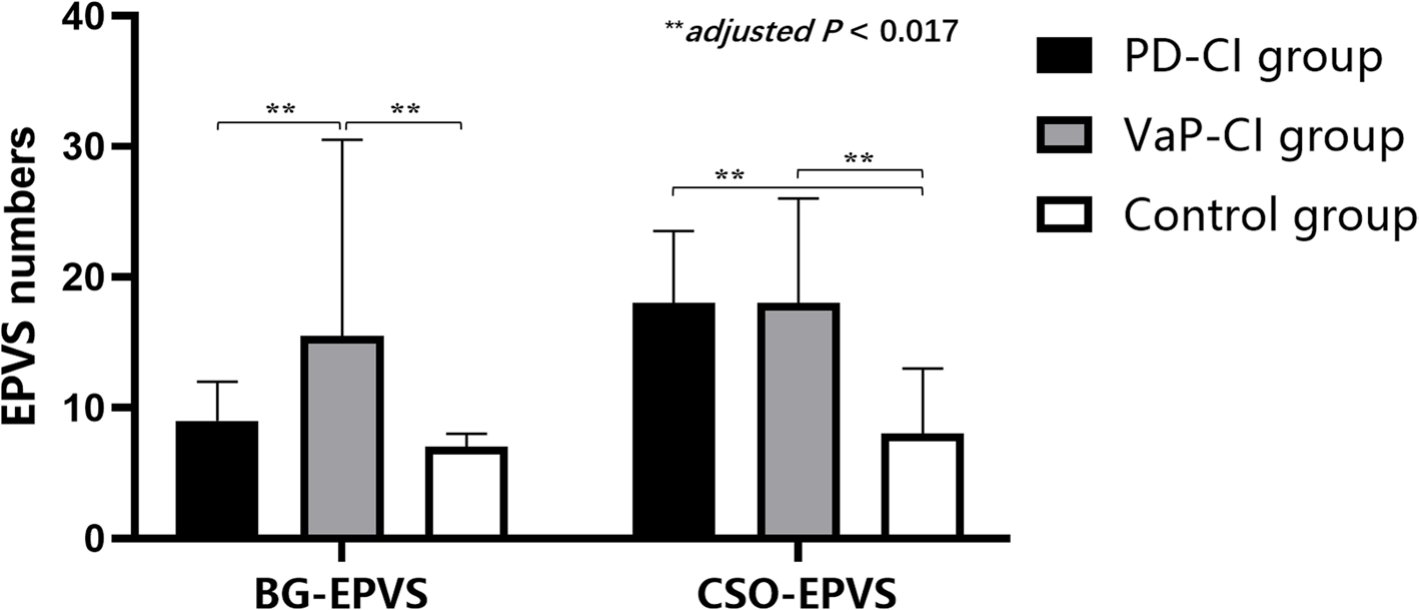
Comparison of the location and number of EPVS among the PD-CI, VaP-CI, and control groups. BG-EPVS in the VaP-CI group were more numerous than in the PD-CI and control groups *(adjusted P* < 0.017). CSO-EPVS in the PD-CI and VaP-CI groups were higher than that in the control group (*adjusted P* < 0.017)

Educational levels in the PD-CI group were higher than those in the PD-NCI group (*P* < 0.05). Lacunes were more frequent in the VaP-CI group than in the VaP-NCI group (*P* < 0.05). The differences in the EPVS, the percentage of patients with EPVS I as the main type, H-Y stages, and UPDRS-III scores of “OFF” between the PD and PD-CI and between the VaP and VaP-CI groups did not reach statistical significance (*P* > 0.05) (Table 2 and Fig. 2).

**Table 2:** Correlation analysis of clinical characteristics with CI and NCI impairment in patients in PD and VaP groups; *M (P25-P75)*

Variable	PD group (*n* = 26)			VaP group (*n* = 19)		
	CI group (*n* = 16)	NCI group (*n* = 10)	*P* values	CI group (*n* = 16)	NCI group (*n* = 3)	*P* values
Age, years	63.00 (58.00–70.75)	62.00 (55.75–64.25)	0.397	75.50 (69.50–80.75)	73.00 (58.00–77.00)	0.341
Education levels: completed secondary school or more, n (%)	10 (62.50)	10 (100)	0.027^*^	9 (56.25)	2 (75.00)	0.737
BG-EPVS numbers	9.00 (7.00–12.00)	7.00 (3.75–15.25)	0.458	15.50 (10.25–30.50)	16.00 (11.00–16.00)	0.779
CSO-EPVS numbers	18.00 (9.50–23.50)	18.50 (10.50–25.25)	0.751	18.00 (7.75–26.00)	18.00 (13.00–18.00)	0.823
EPVS I as the main type, n (%)	3.00 (18.80)	1.00 (10.00)	0.547	9.00 (56.30)	1.00 (33.30)	0.466
Fazekas scores	0.00 (0.00–1.00)	0.00 (0.00–1.50)	0.803	2.00 (1.00–3.75)	1.00 (0.00–1.00)	0.152
GCA scores	1.00 (0.25–2.00)	1.00 (1.00–2.00)	0.825	3.00 (2.00–3.00)	2.00 (2.00–3.00)	0.478
KS scores	2.00 (1.00–3.00)	3.00 (2.00–3.00)	0.117	3.00 (2.25–3.00)	3.00 (2.00–3.00)	0.770
MTA scores	1.00 (0.00–2.00)	1.00 (0.00–1.25)	0.738	2.50 (1.00–3.75)	2.00 (1.00–2.00)	0.358
Lacunes numbers	0.00 (0.00–2.75)	1.00 (0.00–3.50)	0.828	2.00 (0.25–5.50)	0.00 (0.00–0.00)	0.038^*^
CMBs numbers	0.00 (0.00–1.00)	0.00 (0.00–0.00)	0.055	1.50 (1.00–5.75)	0.00 (0.00–2.00)	0.189
UPDRS-III scores “OFF”	42.50 (30.25–45.00)	22.00 (18.50–34.25)	0.086	50.00 (33.50–55.00)	50.00 (30.00–58.00)	0.867
H-Y stages	3.00 (2.00–4.00)	1.00 (2.00–3.00)	0.174	4.00 (3.00–4.75)	3.00 (3.00–4.00)	0.412

^*^P < 0.05

### Difference analysis of clinical characteristics between the PD-CI and VaP-CI groups and the control group

Age, CSO-EPVS, lacunes, Fazekas scores, GCA scores, KS scores, and MTA scores in the PD-CI and VaP-CI groups were higher than those in the control group (*adjusted P* < 0.017). BG-EPVS were more frequent in the VaP-CI group than in the control group (*adjusted P* < 0.017). BG-EPVS, CMBs, Fazekas scores, GCA scores, KS scores, MTA scores, and H-Y stages in the VaP-CI group were higher than those in the PD-CI group (*adjusted P* < 0.017). The difference in age and duration of onset to enrolment between the VaP-CI and PD-CI groups did not reach statistical significance (*adjusted P* > 0.017). The VaP-CI group had higher H-Y stages than the PD-CI group (*P* < 0.05). The differences in the percentage of patients with EPVS I as the main type between the three groups did not reach statistical significance (*adjusted P* > 0.017) (Table 3 and Fig. 2).

**Table 3:** Correlation analysis of clinical characteristics between the PD-CI and VaP-CI groups and control group; *M (P25-P75)*

Variable	PD-CI group (*n* = 16)	VaP-CI group (*n* = 16)	Control group (*n* = 32)	*P* values
Age, years	63.00 (58.00–70.75) ^	75.50 (69.50–80.75) ^	48.50 (37.50–57.00)	< 0.001^***^
BG-EPVS numbers	9.00 (7.00–12.00)	15.50 (10.25–30.50) ^^△^	7.00 (4.00–8.00)	< 0.001^***^
CSO-EPVS numbers	18.00 (9.50–23.50) ^	18.00 (7.75–26.00) ^	8.00 (4.25–13.00)	0.001^**^
EPVS I as the main type, n (%)	3 (18.80)	9 (56.30)	10 (31.30)	0.072
Fazekas scores	0.00 (0.00–1.00) ^	2.00 (1.00–3.75) ^^△^	0.00 (0.00–0.00)	< 0.001^***^
GCA scores	1.00 (0.25–2.00) ^	3.00 (2.00–3.00) ^^△^	0.00 (0.00–0.00)	< 0.001^***^
KS scores	2.00 (1.00–3.00) ^	3.00 (2.25–3.00) ^^△^	0.00 (0.00–0.00)	< 0.001^***^
MTA scores	1.00 (0.00–2.00) ^	2.50 (1.00–3.75) ^^△^	0.00 (0.00–0.00)	< 0.001^***^
Lacunes numbers	0.00 (0.00–2.75) ^	2.00 (0.25–5.50) ^	0.00 (0.00–0.00)	< 0.001^***^
CMBs numbers	0.00 (0.00–1.00)	1.50 (1.00–5.75) ^#^	-	0.002^**^
UPDRS-III scores “OFF”	42.50 (30.25–45.00)	50.00 (33.50–55.00)	-	0.076
H-Y stages	3.00 (2.00–4.00)	4.00 (3.00–4.75) ^#^	-	0.031^*^
Duration of onset to enrolment, years	1.00 (0.50–2.75)	3.50 (1.25–6.00)	-	0.155

^*^P < 0.05, ^**^P < 0.01, ^***^P < 0.001. Compared to the control group, ^*adjusted P* < 0.017. Compared to the PD-CI group, ^△^*adjusted P* < 0.017, ^#^*P* < 0.05

### Logistic regression analysis of clinical characteristics of PD-CI and VaP-CI patients

The PD-CI group and VaP-CI group were used as classification dependent variables, and clinical characteristics that were significantly different between the groups in Table 3 were included in the multivariate logistic regression analysis, including BG-EPVS numbers, Fazekas scores, GCA scores, KS scores, MTA scores, CMB numbers, and H-Y stages. GCA scores, KS scores, and MTA scores all represent brain atrophy, so the brain atrophy scores are equal to the sum of GCA scores, KS scores, and MTA scores. BG-EPVS numbers, Fazekas scores, brain atrophy scores, CMB numbers, and H-Y stages were used as independent variables for multivariate logistic regression analysis. However, none of these clinical characteristics was statistically significant (*P* > 0.05) (Table 4).

**Table 4:** Logistic regression analysis of clinical characteristics of PD-CI and VaP-CI patients

Variable	Odds ratio (95% CI)	*P*
BG-EPVS numbers	1.27 (0.92–1.76)	0.141
Fazekas scores	0.76 (1.86–3.08)	0.697
Brain atrophy scores	3.00 (0.64–14.11)	0.163
CMBs numbers	12.02 (0.49–293.44)	0.127
H-Y stages	3.65 (0.33–40.53)	0.292

PD-CI group and VaP-CI group were used as classification dependent variables. Clinical characteristics that were significantly different between the groups in Table 3 were included in multivariate logistic regression analysis. GCA scores, KS scores, and MTA scores all represent brain atrophy, so the brain atrophy scores are equal to the sum of GCA scores, KS scores, and MTA scores. BG-EPVS numbers, Fazekas scores, brain atrophy scores, CMB numbers, and H-Y stages were used as independent variables for multivariate logistic regression analysis. None of these clinical characteristics reached statistical significance, *P* > 0.05

### ROC curves of BG-EPVS, Fazekas, and GCA Scores for VaP-CI occurrence

**Fig. 3:**
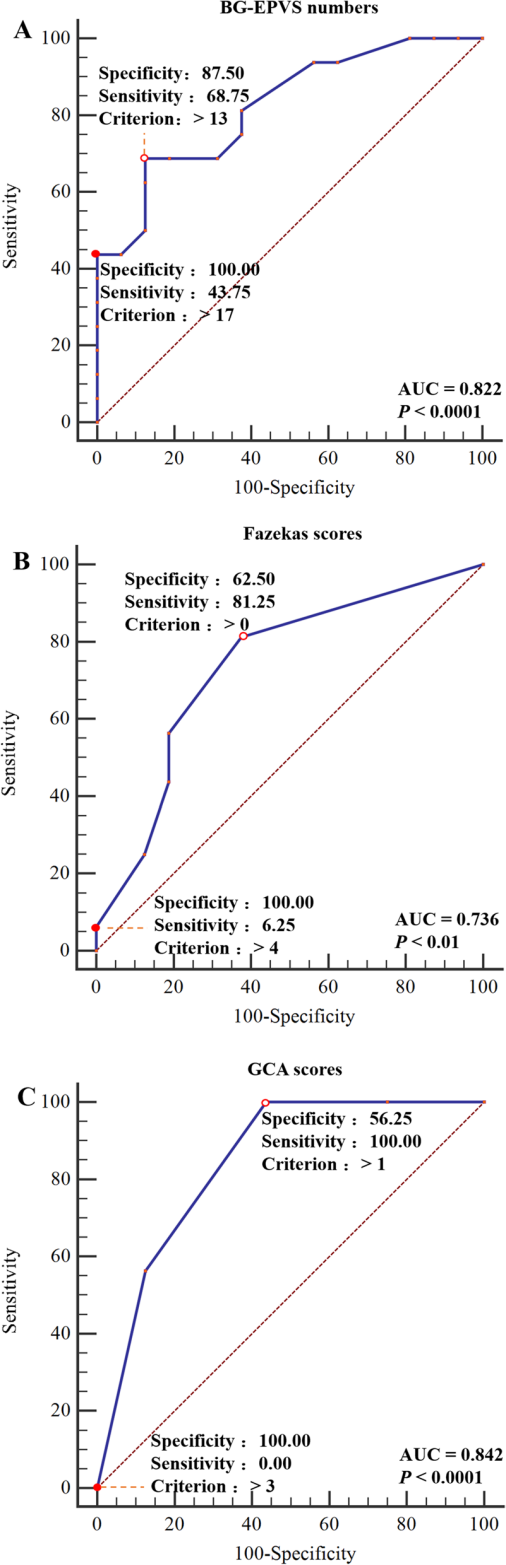
ROC curves of BG-EPVS, Fazekas scores, and GCA scores for VaP-CI occurrence. **A** BG-EPVS numbers, **B** Fazekas scores, **C** GCA scores. The PD-CI group was used as the reference group, and the VaP-CI group was used as the measurement group.  The diagnostic cutoff value corresponds to the maximum point of the Youden index.  The diagnostic cutoff value corresponds to the maximal specificity

ROC curve analyses were performed with the VaP-CI group as the trial group and the PD-CI group as the control group. We found that the differences in BG-EPVS and Fazekas and GCA scores were statistically significant (*P* < 0.01). The cutoff value was determined by the maximum Youden index. The best cutoff value for BG-EPVS was 13, which achieved a specificity of 87.50% and a sensitivity of 68.75% (95% confidence interval (95% CI): 0.647–0.934). When the cutoff was greater than 17, BG-EPVS had a sensitivity of 43.75% and a specificity of 100.00% (Fig. 3: A). The best cutoff value for Fazekas scores was 0, which achieved a specificity of 62.50% and a sensitivity of 81.25% (95% CI: 0.551–0.876). When the cutoff was greater than 4, Fazekas scores had a sensitivity of 6.25% and a specificity of 100.00% (Fig. 3: B). The best cutoff value for GCA scores was 1, which achieved a specificity of 56.25% and a sensitivity of 100.00% (95% CI: 0.670–0.946). When the cutoff was greater than 3, GCA scores had a sensitivity of 0.00% and a specificity of 100.00% (Fig. 3: C). The presence of BG-EPVS was accepted as the best indicator for assessing the PD-CI and VaP-CI groups.

## Discussion

We found that CSVD imaging findings, such as EPVS, WMH, lacunes, and brain atrophy, were common in patients with VaP and VaP-CI, especially the EPVS in the basal ganglia, which was different from the findings in patients with PD and PD-CI.

### Difference analysis of EPVS, Lacunes, WMH, brain atrophy, and CMBs between PD and VaP

Multiple case reports have shown that the severity of BG-EPVS may be related to the occurrence of Parkinson’s symptoms and extrapyramidal symptoms and a decline in cognitive function [17]. Burnett et al. [6] found that striatal EPVS were not correlated with the development of extrapyramidal clinical diseases, including PD. The present study showed that the increase in the numbers of EPVS and lacunes and the increases in the severity of WMH and brain atrophy were both high-risk factors for PD and VaP and were more severe in VaP patients. For PD, the number of CSO-EPVS was increased, while for VaP, the numbers of BG-EPVS, CSO-EPVS, and CMBs were increased. CSO-EPVS are closely correlated with degenerative diseases, while BG-EPVS are correlated with vascular disease factors. Considering that PD is a degenerative disease, the EPVS imaging features in PD were mainly CSO-EPVS, as shown in the imaging results. Kim et al. [18] found that the incidence of CMBs in VaP patients (56%) was significantly higher than that in PD patients (17.7%), and similar results were obtained in the present study. CMBs are one of the imaging manifestations of CSVD, and the pathological mechanism of CMBs is currently believed to be hyaline degeneration of small blood vessels caused by hypertension or atherosclerosis. In summary, the imaging manifestations of CSVD were more severe in VaP patients and were closely correlated with atherosclerosis. Since VaP and PD may have common vascular risk factors, differentiation between these diseases requires assistance from more biomarkers and imaging markers.

### Difference analysis of EPVS, Lacunes, WMH, brain atrophy, and CMBs between PD-CI and VaP-CI

Shibata et al. [19] found that paraventricular WMH, BG-EPVS, and brain atrophy were predictors of PD-CI. Meanwhile, Ham et al. [20] showed that CMBs were more common in PD-CI patients than in PD-NCI patients. The present study found that the numbers of CSO-EPVS and lacunes and the severity of WMH and brain atrophy in the PD-CI group were all higher than those in the control group. However, in the comparison between the PD-NCI and PD-NCI groups, no differences in the number and location of EPVS, the number of lacunes and CMBs, or the severity of brain atrophy and WMH were found, which is different from previous studies. The above results indicate that the imaging manifestations of CSVD are common in PD-CI patients, but they are not the key to whether PD patients have CI and cannot be used as predictors of the occurrence of PD-CI. Because the diagnosis of PD is more complicated and PD usually occurs in the elderly population, more vascular factors and pathological changes in Alzheimer’s disease (AD) might be included, thus affecting the results.

VaP patients often have CI, especially the occult subtype. Brain atrophy, WMH, and lacunes are common in VaP-CI [21]. The VaP patients enrolled in the present study all presented with the occult subtype, and the incidence of VaP-CI was 84.21%. No differences were found between the VaP-CI and VaP-NCI groups in the number and location of EPVS, Fazekas score, brain atrophy, and the number of CMBs, and a difference only in the number of lacunes was observed. In the VaP-CI group, more vascular risk factors and a greater CSVD vascular load were observed; however, for the relationship with the timing and induction of the onset of VaP-CI, the current observations showed no significant correlations with EPVS, WMH, brain atrophy, and CMBs. The high-risk factors driving this transformation require further studies and follow-ups with large sample sizes.

Studies have found that VaP patients have more severe CI than PD patients [22]. Few reports are available on the difference between VaP-CI and PD-CI patients in CSVD imaging results. This study found that the numbers of BG-EPVS and CMBs, Fazekas scores, GCA scores, KS scores, and MTA scores were higher in the VaP-CI group than in the PD-CI group. Meanwhile, there was no significant difference in age between PD-CI and VaP-CI. The above results reveal that when comparing VaP-CI with PD-CI patients, age differences were excluded, manifestations were mostly CSVD factors rather than neurological degeneration and degenerative diseases, and EPVS were more concentrated in the BG. The possible mechanism is as follows: subcortical and deep BG represent important pathways for many nerve fibre bundles, and the occurrence of the abovementioned lesions leads to disruption of the subcortical circuit of the frontal lobe, especially the left inferior fronto-occipital fasciculus (IFOF) and the right inferior longitudinal fasciculus (ILF) [23], leading to the occurrence and progression of CI [24]. The increased numbers of BG-EPVS and CMBs and the increased severity of WMH and brain atrophy can be used as auxiliary imaging markers to distinguish between VaP-CI and PD-CI. The logistic regression of this study found that BG-EPVS, WMH, brain atrophy, and CMBs could not be used as independent factors. Because EPVS often occur with WMH or brain atrophy, BG-EPVS cannot be used as an imaging marker alone. Although the disease duration in the PD-CI and VaP-CI groups was not completely homogeneous (1.00 year (0.50–2.75) and 3.50 years (1.25–6.00)), and there was no statistical significance between the two groups, we believe that the above conclusion is more appropriate for VaP-CI and PD-CI patients respectively in the first 3.5 and 1 years on average from onset to enrolment. Subsequent studies will supplement this study's data on the duration of onset to enrolment of the disease with more patients enrolled.

The diagnosis of VaP is sometimes prone to errors, i.e., VaP may represent pseudovascular parkinsonism (such as PD and PSP); therefore, pathological or effective biomarkers are needed. In this study, the ROC curve analysis showed that when the number of BG-EPVS was > 17, the Fazekas scores were > 4, or the GCA scores were > 3, the specificity of the diagnosis of VaP-CI was the highest, which can be used as an auxiliary diagnostic indicator for VaP-CI patients.

The limitations of this study are as follows: 1. Failure to conduct DAT-PET-CT detection cannot exclude the comorbidity of VaP and PD. 2. The insufficient follow-up resulted in failure to dynamically monitor changes in CI in VaP and PD patients.

In summary, this study showed that increased severity of EPVS, lacunes, WMH, and brain atrophy were both high-risk factors for PD, VaP, and cognitive impairment, especially in patients with VaP and cognitive impairment. The increases in the number of BG-EPVS and CMBs and in the severity of WMH and brain atrophy were more closely correlated with VaP-CI, which can be used as imaging markers for the differentiation of VaP-CI and PD-CI, providing more evidence for clinical differential diagnosis.

## Data Availability

All data generated or analysed during this study are included in this article. Further enquiries can be directed to the corresponding author.

## Abbreviations

PD: Parkinson’s disease
VaP: Vascular parkinsonism
EPVS: Enlarged perivascular spaces
BG: Basal ganglia
CSO: Centrum semiovale
CI: Cognitive impairment
CSVD: Cerebellar small vessel disease
GCA: Global cortical atrophy
KS: Koedam posterior atrophy visual scale
MTA: Medial temporal atrophy
WMH: White matter hyperintensities
